# The Prevalence and Correlates of Breast Cancer among Women in Eastern China

**DOI:** 10.1371/journal.pone.0037784

**Published:** 2012-06-18

**Authors:** Zhi-Gang Yu, Cun-Xian Jia, Li-Yuan Liu, Cui-Zhi Geng, Jin-Hai Tang, Jin Zhang, Qiang Zhang, Yu-Yang Li, Zhong-Bing Ma

**Affiliations:** 1 Department of Breast Diseases, the Second Hospital of Shandong University, Jinan, China; 2 Epidemiology Institute, School of Public Health, Shandong University, Jinan, China; 3 Department of Sociology, State University of New York College at Buffalo, Buffalo, New York, United States of America; 4 Department of Breast Diseases, the Fourth Hospital of Hebei Medical University, Shijiazhuang, China; 5 Department of Breast Diseases, Jiangsu Cancer Hospital, Nanjing, China; 6 Department of Breast Diseases, Tianjin Cancer Hospital, Tianjin, China; Pontificia Universidad Catolica de Chile, Faculty of Medicine, Chile

## Abstract

The purpose was to investigate the prevalence rate, characteristics and related factors of breast cancer among women in Eastern China. A total of 122,058 female subjects completed the study, with 320 confirmed cases of breast cancer (crude prevalence: 262.5/100,000; standardized prevalence: 207.7/100,000). Among all of the identified breast cancer cases, 91.6% were diagnosed after the age of 35 and 60.0% were diagnosed before menopause. The odds ratios (95% confidence interval) of those breast cancer risk factors as selected through multivariate logistic regression were as follows: 5.438 (1.553–19.004) for family history of breast cancer, 3.556 (1.880–6.728) for high behavior intervention score, 3.556 (0.904–13.994) for history of diabetes, 3.357 (1.131–9.969) for history of benign breast tumors, 2.196 (1.355–3.556) for poor overall life satisfaction, 1.826 (0.995–3.350) for premenopause of breast cancer, 1.528 (1.083–2.155) for high BMI index, 1.500 (0.920–2.446) for poor financial status, 1.497 (1.014–2.211) for multiple miscarriages/abortions, and 1.231 (0.972–1.559) for infrequent consumption of garlic (frequent garlic consumption is a protective factor). There were significantly more cases of breast cancer diagnosed prior to menopause than after menopause, and most of the patients were diagnosed after the age of 35. These findings suggest that attention should be focused on the incidence of breast cancer among premenopausal women older than 35.

## Introduction

Breast cancer is one of the most common malignancies in women worldwide and is the leading cancer-related cause of death in women [1], [2]. Since the 1970s, there have been no population-based large-scale surveys or nationwide disease survey except some hospital-based study for breast cancer in China. Some regional surveys have indicated that the incidence of breast cancer is rising in Chinese women [3]. However, most of these studies were focused on economically–developed, large cities, such as Beijing, Shanghai and Tianjin, despite nearly 70% of Chinese population being located in rural areas. Substantial international and domestic research has focused on the influencing factors associated with breast cancer. However, these studies were largely based on clinical cases with limitations due to small sample size, different ethnic groups within the target population and differences in regional social-economic conditions and customs, which resulted in inconsistent conclusions. In general, the causes of breast cancer are still not fully understood; therefore, obtaining relevant risk factors for breast cancer through a population-based case-control study can contribute to the effective prevention and intervention of breast cancer among the population and make significant contributions to public health. In this study, a cross-sectional survey was performed in Eastern China between July 15 and September 15, 2008, to better understand the prevalence rate, characteristics and risk factors for breast cancer in Chinese women.

## Methods

### Study Subjects

**Figure 1:**
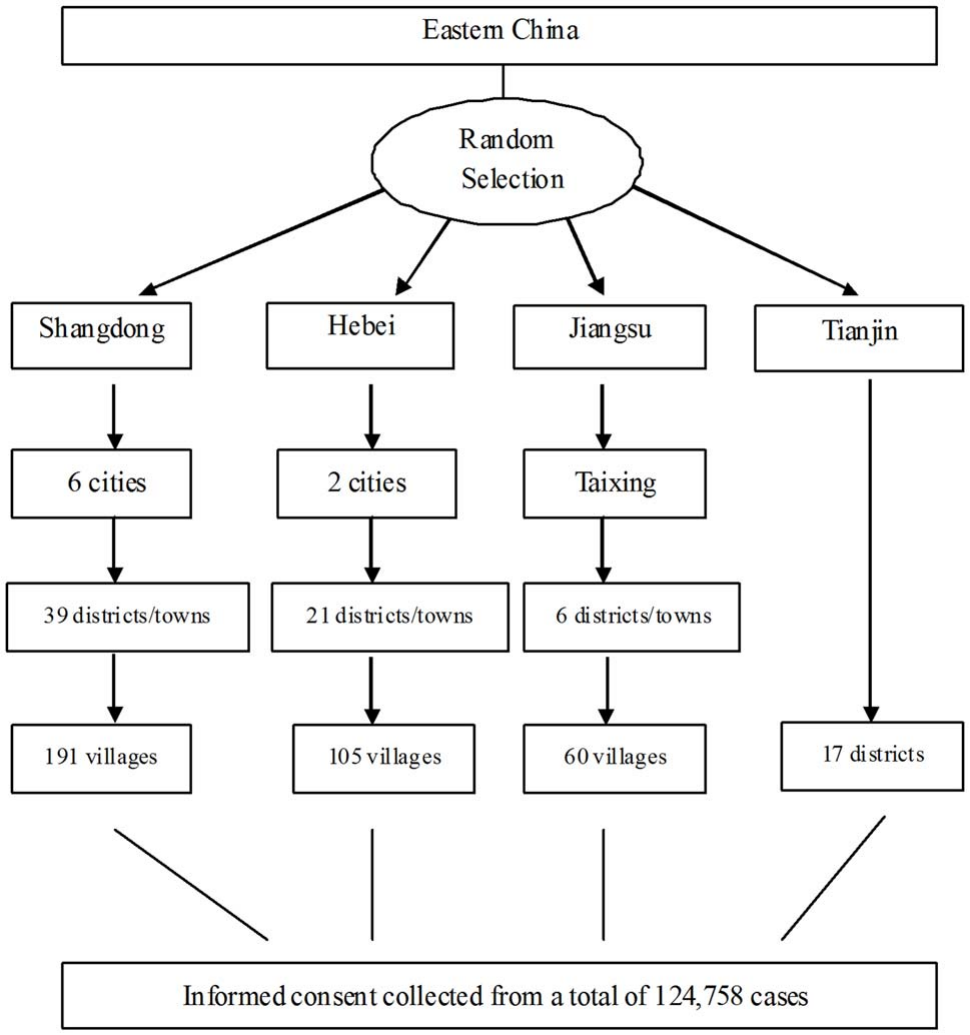
Random samples were obtained through multi-stage stratified cluster sampling. Shandong, Jiangsu, Hebei and Tianjin were selected as the sampling provinces because these provinces were manliy resident areas of Han ethnic group. And counties/regions and villages/communities were subsequently randomly selected from each province. Totally, 124,758 female, 1.5 times of the required sample size, were selected as study population. And the details of sampling information were shown in this figure.

Random samples were obtained through multi-stage stratified cluster sampling. The target population included 25- to 70-year-old females of the Han ethnic group with over two years of local residence and at least six months of local residence at the time of survey. Long-term migrant workers, who left hometown for work, were excluded from this study. The provinces in Eastern China where the Han ethnic group mainly resides, including Shandong, Jiangsu, Hebei and Tianjin, were selected as the survey provinces. Subsequently, counties or regions were randomly selected from each province. Finally, villages or communities were randomly selected from the sampled counties or regions, and women who met the study requirements were selected for the survey. The required sample size of 86,667 subjects was estimated using a Poisson distribution with 300 cases expected and an estimated prevalence rate of 200/100,000 [4]–[6]. Because cluster sampling was employed, the actual sample size needed was considered to be 1.5 times of the required sample size; thus, 130,000 subjects were needed for the survey. The details of the sampling procedure are shown in Figure 1.

Those breast cancer patients (123 cases) who were diagnosed within two years of the survey were selected as the breast cancer group, and the nearest neighbors of the patients, who were healthy and had no blood relationship with the patients, were selected as the control group in accordance with the 1∶3 principle. The matching factors were age (same age ±2 years) and location (neighbor or co-worker in the same region).

### Questionnaire Content

Data were collected through in-person interviews based on a self-designed structured questionnaire. The questionnaire included six aspects gathered from patient interviews: (i) demographic characteristics: age, marital status, education, occupation, household income, height, body weight, financial status and social status; (ii) female physiological and reproductive factors: age at menarche, age at menopause, menstrual cycle history, childbearing history, breastfeeding methods, abortions or miscarriage, contraceptive methods and use of contraceptive medicine; (iii) medical and family history: primarily breast-related diseases and family history of breast cancer; (iv) dietary habits: frequency of the intake of various types of food; (v) lifestyle habits: smoking (including passive smoking), alcohol intake, tea intake, physical exercise and mental and psychological conditions (the items under psychological status were summed to calculate the overall life satisfaction and current life satisfaction scores); (vi) breast-cancer-related knowledge: risk factors for breast cancer and early signs and symptoms of disease (the cumulative scores of these relevant items were counted as the related knowledge score and behavioral prevention score). The last component of the questionnaire was gathered from the clinical breast exam and included the results from visual examination, palpation and related diagnostic tests.

With the exception of the basic demographic information, all questions had multiple-choice responses and attempts were made to quantify or categorize the answer choices (e.g., yes/no or 1/2/3/4). For all of the variables covered by the questionnaire, the answers were defined by strict criteria (See Table 1) [4].

**Table 1:** Explanation of terms used in the questionnaire.

Terms	Explanation
Household member	The actual household members who are residing and living together.
Employment	The recent employment or the longest employment in the past year.
Body mass index (BMI)	The body height, weight, waist circumference, and hip circumference were measured on site, and then BMI was calculated.
Times of pregnancy and delivery	All the pregnancy times, regardless of the outcomes including live birth, stillbirth, spontaneous abortions, and artificial abortion.
History of breast-feeding	The actual breast-feeding duration (unit: months) was recorded.
Menstrual history	Based on NCCN 2008 definition.
Relevant diseases	Only formally confirmed diseases were included.
Smoking	At least one cigarette per day for at least one year.
Second-hand smoke	Whether there is any person who live or work with you smokes.
Insomnia	Difficulty falling asleep or remaining asleep, poor sleep quality, and remarkably reduced sleep time, which is harmful for health and affects the normal daily activities.
Wake up early and stay up late	Wake up 2 hours earlier in the morning or stay up 2 hours later at night when compared with the normal sleep-wake schedule.
Diets and alcohol	Based on the consumption frequency, the diets and alcohol was measured suing 1/2/3/4 scale.
Life satisfaction	The overall life satisfaction (current, future, and past) and the current life satisfaction (on housing, income, health, marriage, medical care, and neighbors) were measured using 1/2/3/4/5 scale.
Hormone replacement	The hormone refers to the natural estrogen (estradiol, estrone, and estriol) and synthetic estrogens.
Drug history	Medication hsitory was recorded once a drug was used (unit: months).

### Implementation

**Table 2:** Criteria for suspected positive physical signs in breast exam.

	Physical signs
**1**	Palpation of irregular mass
**2**	Nipple discharge (including bloody, yellow-green and colorless discharge)
**3**	Nipple retraction and changes in surrounding skin (including peau d’orange, dimple sign, erythema and edema)
**4**	Swollen or hard axillary lymph nodes

**Figure 2:**
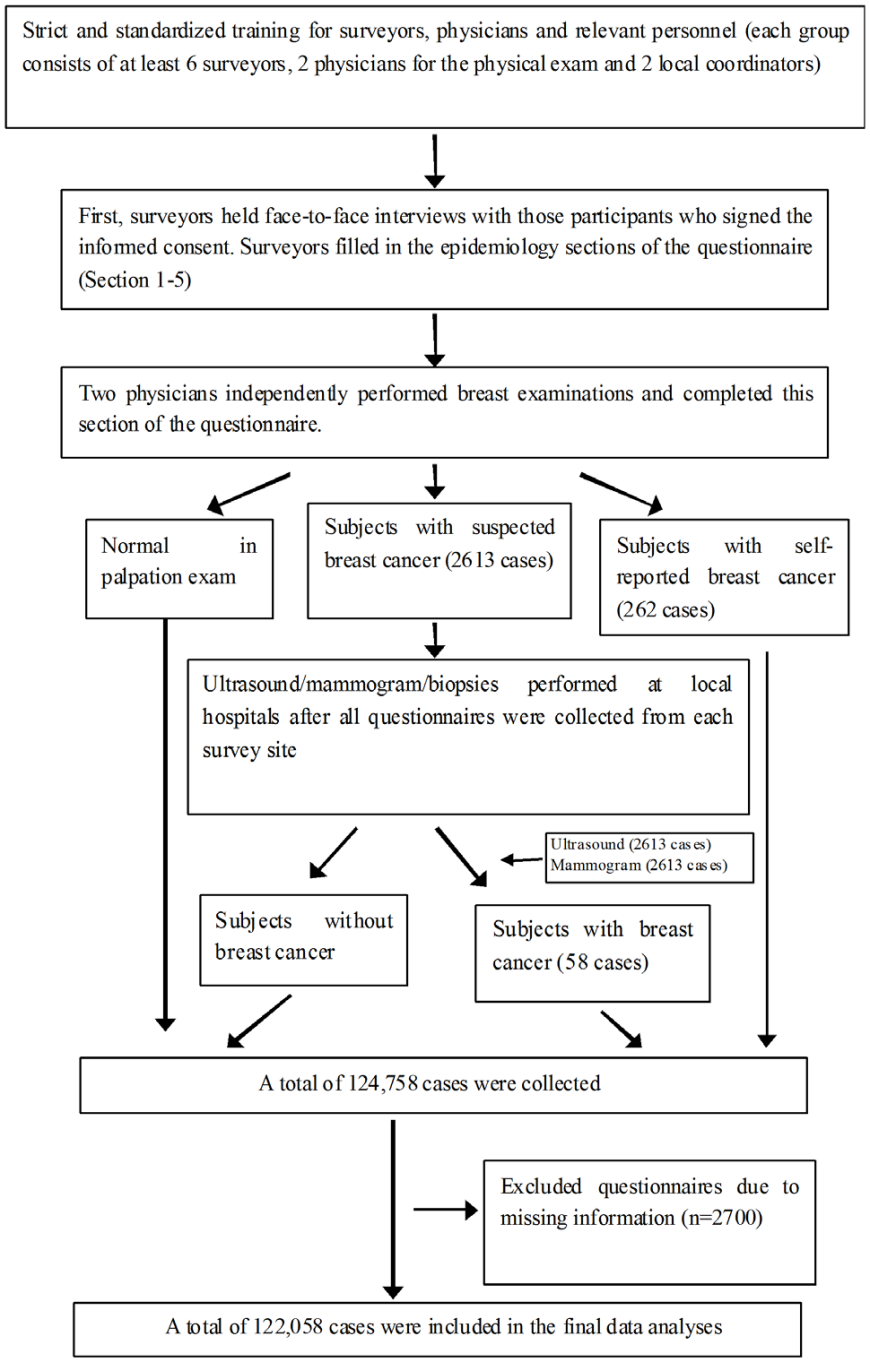
The investigation involved face-to-face interviews based on self-designed structured questionnaires and subsequente clinical breast examinations. And subjects with suspected positive physical signs would receive further ultrasonic and X-ray examinations and possible biopsies. After the above proceeds, breast cancer cases were identified. In total, 124,758 were collected in the study, and because of information loss, 122,058 cases were included in the final analysis.

This study was approved by the ethics committees at the collaborating institutions in each region and informed consent was obtained from all the study subjects. Investigators and physicians were recruited in each survey region before the implementation of this study. To ensure objectivity and authenticity, all participating investigators and physicians performing physical examinations received strict and standardized professional training before the implementation of the project. The main on-site data collection procedure is shown in Figure 2. The clinical breast examination consisted of two components: a basic breast examination (including visual examination and palpation) and standardized auxiliary diagnostic test, such as an ultrasound and/or mammogram, for survey subjects with suspected disease on physical exam (the detailed criteria for suspected positive physical signs of disease are shown in Table 2). The subjects diagnosed with breast cancer on biopsy were enrolled into the breast cancer group, while healthy subjects, as confirmed by clinical physical examination and diagnostic imaging, were included in the control group. A total of 2,613 subjects had an abnormal breast examination, all of who received an ultrasound and mammogram. Among these subjects, 58 had abnormalities detected with diagnostic imaging, and 262 reported being previously diagnosed with breast cancer.

**Figure 3:**
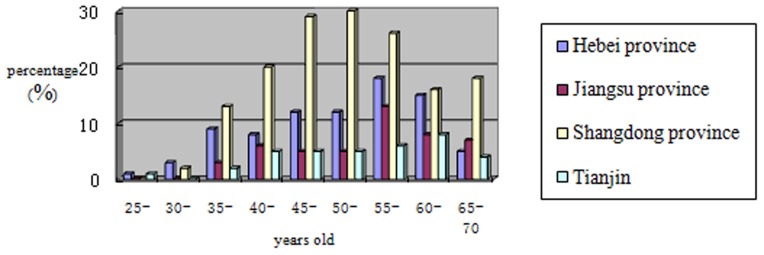
Detailed distributions of the breast cancer cases from the four survey areas by age are shown in this figure. Overall, breast cancer cases identified in this study were primarily diagnosed in the 40–45 and 45–50 year age groups.

**Table 3:** Pathological information on cases of breast cancer.

	Number of cases (n)	Percent (%)
**Pathological type**		
** Invasive cancer**	179	81
** Non-invasive cancer**	20	9.05
** Unknown**	22	9.95
**OR-status(oestrogen receptor)**		
** Positive**	76	34.3
** Negative**	20	9.05
** Unknown**	125	56.6
**Lymph node status**		
** Positive (1–4)**	49	22.2
** Positive (>4)**	23	10.4
** Negative**	94	42.5
** Unknown**	55	24.9
**Tumour size**		
** <2 cm**	22	9.95
** 2 cm–5 cm**	97	43.9
** >5 cm**	19	8.6
** Unknown**	83	37.6
**Clinical stage**		
** 0**	18	8.14
** I**	21	9.5
** II**	92	41.6
** III**	24	10.9
** IV**	1	0.5
** Unknown**	65	29.4

To test the reliability of the clinical breast examination performed for this study, 145 subjects were randomly selected to form a normal control group. All the subjects in this control group were randomly selected from the survey subjects with a normal physical exam. This group then received an ultrasound and mammogram. Two radiologists interpreted the ultrasound and mammograms based on Breast Imaging-Reporting and Data System (BI-RADS) classification [5]. After group consultation with the specialists, 123 subjects (84.83% of 145) were classified as BI-RADS 1, 14 (9.66%) as BI-RADS 2 and 8 (5.52%) as BI-RADS 3, which suggested that the physical examination results in this study were reliable.

### Quality Control

All interviews and clinical examinations in this survey were completed within two months, and all survey subjects were selected strictly according to the random sampling method. During the survey process, a supervisor in each region oversaw the surveyors and sampled their work for inspection. Further, 10% of the questionnaires completed at the end of each day were randomly sampled to inspect for their completeness, accuracy and standardization. After the quality assessment was completed, all identified errors and missed survey items were promptly corrected. If some of information were missed in the questionnaire, the interviewer would collect the missing information from the original survey site the next day. Finally, after the completion of survey, sampled re-screening was performed for each survey site, and the results showed a consistency of 92.19%. Clinical breast examinations were performed independently on each survey subject by two physicians who had received standardized training and had over three years of working experience in a breast surgery department. When the examination results from the two physicians were inconsistent, the supervisor at the survey site would discuss the case with the two physicians and make a final decision. Standardized training was provided to the personnel who performed data entry and double data entry was performed.

### Statistical Analyses

The database was established using the software EpiData3.1. Statistical methods, including t-test, χ^2^ test, and univariate and multivariate conditional logistic regression analyses, were used to identify the risk factors for breast cancer. The odds ratios (OR) with 95% confidence intervals were also calculated. All data analyses were performed using SPSS16.0.

## Results

**Figure 4:**
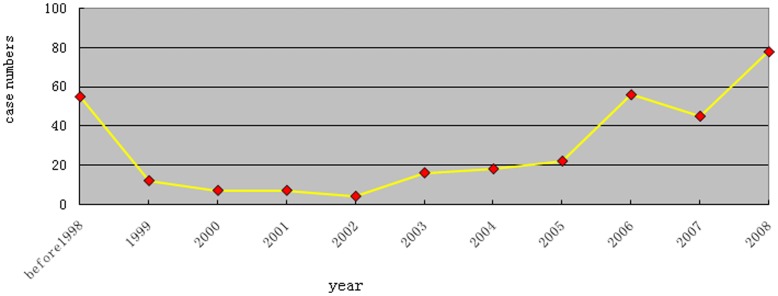
The prevalence of breast cancer varied with significantly over time: among the 320 breast cancer cases, 179 (55.94%) were diagnosed within the past three years, including 78 cases within the last year, 45 cases within the last two years and 56 cases within the last three years.

**Table 4:** Related factors of demographic variables of breast cancer.

Variables	Number of cases	participants	The prevalent rate (1/100,000)	χ^2^	*p*
Location					
Urban	84	33696	249.3	0.295	0.287
Rural	236	88362	267.1		
Education					
Elementary	154	48570	317.1	15.859	0.001
Middle	96	42483	226.0		
High	55	19186	286.7		
College or above	15	11639	128.9		
Personal annual income (RMB)					
<10000	223	77858	286.4	7.859	0.005
> = 10000	68	34907	194.8		
Marriage					
No	1	1437	69.6	2.062	0.151
Yes	319	120621	264.5		
Body Mass Index					
<25	186	91488	203.3	55.667	0.000
25−	111	27075	410.0		
> = 30	23	3497	657.7		
Family history of breast cancer					
Yes	21	1120	1875.0	112.400	0.000
No	299	120938	247.2		

In this survey, 147,538 women were initially selected, and of these women, 124,758 were reached to complete the survey, giving a rate of loss at 15.44%. Of the 124,758 women surveyed, 122,058 were included in the final analysis because 2,700 questionnaires had to be excluded due to incomplete information.

**Table 5:** Related factor of physiological variables to breast cancer.

Variables	Cases	Participants	Theprevalentrate	χ^2^	P
Age of menarche					
7–11	3	867	346.0	1.782	0.619
12–13	43	19395	221.7		
14 or more	274	101744	269.3		
Missing value	0	52	0		
Regularity of menstruation					
Yes	291	114514	254.1	4.959	0.032
No	29	7544	384.4		
Parity					
<2	118	70719	166.9	58.414	0.000
> = 2	202	51339	393.5		
Times of misbirth					
0	199	91433	217.6	e	0.000
1	75	20632	363.5		
2	34	7398	459.6		
>2	12	2427	494.4		
Dysmenorrheal					
No	253	100313	252.2	2.136	0.144
Yes	67	21745	308.1		
Menopause or stop					
No	122	85755	142.3	158.5	0.000
Yes	198	36303	545.4		
Oral contraceptive					
Yes	18	5580	322.6	1.180	0.554
No	298	114061	261.3		
Missing value	4	2101	190.4		
Benign of breast gland tumor					
Yes	27	854	3161.6	276.6	0.000
No	293	121204	241.7		
Hyperplasia of galactophore					
Yes	31	7487	414.0	7.037	0.008
No	289	114571	252.2		

**Table 6:** Association between chronic diseases and breast cancer.

variables	Cases	Participants		The prevalentrate (/1 million)		χ^2^			P
Cervix Cancer									
Yes	3	274		1094.9		7.283			0.007
No	317	121784		260.3					
Ovary Cancer									
Yes	0	43		0		0.113			0.737
No	320	122015		262.3					
Ovary cyst									
Yes	9	1488		604.8		6.764			0.009
No	311	120570		257.9					
Diabetes mellitus									
Yes	16	1505		1063.1		37.385			0.000
No	304	120553		252.2					
Hypertension									
Yes	42	7467		562.5		27.431			0.000
No	278	114591		242.6					
Coronary heart disease									
Yes	9	1701		529.1		4.701			0.030
No	311	120357		258.4					
Nephritis									
Yes	2	221		905.0		3.499			0.061
No	318	121837		261.0					
Mental disorders									
Yes	1	148		675.7		0.969			0.325
No	319	121910		261.7					

**Table 7:** The basic demographic information of the patient and control groups.

Variables	Case N (%)	Control N (%)	χ^2^(df)	p
Residence				
Urban	27 (22.0)	97 (26.3)	0.920 (1)	0.337
Rural	96 (78.0)	272 (73.7)		
Education				
Elementary or low	58 (47.2)	192 (52.0)	4.696 (3)	0.195
Middle	36 (29.3)	86 (23.3)		
High	24 (19.5)	60 (16.3)		
College	5 (4.0)	31 (8.4)		
Economic statues				
Very good(1)	4 (3.3)	10 (2.7)	7.858 (3)	0.049
Good(2)	15 (12.2)	76 (20.6)		
Normal(3)	92 (74.8)	266 (72.1)		
Poor(4)	12 (9.8)	17 (4.6)		
Social statues				
Very good(1)	4 (3.3)	11 (3.0)	9.607 (3)	0.022
Good(2)	18 (14.6)	77 (20.9)		
Normal(3)	93 (75.6)	275 (74.5)		
Poor(4)	8 (6.5)	6 (1.6)		
Family annual income (RMB)				
<15000	69 (56.1)	178 (48.2)	2.279 (1)	0.131
> = 15000	54 (43.9)	191 (51.8)		
Marriage				
Never	5 (4.1)	19 (5.1)	0.234 (1)	0.629
Ever	118 (95.9)	350 (94.9)		
Body Mass Index				
<24.0	57 (46.3)	206 (55.9)	9.852 (3)	0.020
24.0–28.0	34 (27.6)	111 (30.1)		
> = 28.0	32 (26.0)	52 (14.1)		
Family history of breast cancer				
Yes	10 (8.1)	7 (1.9)	10.744 (1)	0.001
No	113 (91.9)	362 (98.1)		

The average age of the 122,058 surveyed women was 44.2 years old (SD = 11.6). There were 19,741 subjects (16.2%; M = 42.8 years old) from Hebei Province, 20,921 subjects (17.1%; M = 46.3 years old) from Jiangsu Province, 61,104 subjects (50.1%; M = 43.50 years old) from Shandong Province and 20,292 subjects (16.6%; M = 45.6 years old) were from Tianjin. A total of 88,362 surveyed subjects were from rural areas and 33,696 were from urban areas. The ratio of urban to rural population was 0.38∶0.62, which is similar to the data from the census in China in 2000 (0.36∶0.64). Among the surveyed population, 85,752 were premenopausal (70.3%) and 36,306 (29.7%) were postmenopausal. The average age at menopause in the postmenopausal subjects was 48.8 years old (SD = 4.11). The average age at menarche in all surveyed women was 15.4 years old (SD = 2.0). There were 118,623 (97.2%) subjects who had at least one child before the survey and 95,434 (78.6% of 118,623) who had breast-fed for at least 12 months.

This study found 320 cases of breast cancer, with a prevalence rate of 262.5/100,000 and a standardized prevalence rate of 207.7/100,000 (adjusted based on the 2007 China’s National Population Age Structure). Among these breast cancer cases, 58 (18.12%) were newly identified, 265 (82.81%) were diagnosed within the last 10 years and 55 (17.19%) were diagnosed more than 10 years ago. The mean age of the patients with breast cancer was 47.8 years old (SD = 9.3),293(91.6%) were diagnosed after the age of 35 and 27(8.4%) were diagnosed before the age of 35, This group included 122 premenopausal patients (38.12%) and 198 postmenopausal patients (61.88%). The average age at diagnosis was 46.8 years old (SD = 9.3), and 192 (60%)of the subjects were premenopausal, while 128(40%) were postmenopausal. The average age of menopause for the postmenopausal breast cancer patients was 48.4 years old (SD = 4.5). The average menarche age of the breast cancer patients was 15.8 years old (SD = 2.2). Among the 320 patients, 278 (88.3%) had breast-fed for at least 12 months. Pathologic diagnosis was performed in 221 (69%) cases, with 81%(179/221) of these patients diagnosed with invasive breast cancer. The detailed pathology results are shown in Table 3.

The crude prevalence rate of breast cancer varied in different regions: 420.5/100,000 (366.9/100,000) in Hebei Province, 252.1/100,000 (205.5/100,000) in Shandong Province, 224.7/100,000 (151.9/100,000) in Jiangsu Province and 178.7/100,000 (133.5/100,000) in Tianjin. Standardized prevalence rates based on the age data of 2007 China’s national population is shown in parentheses. Detailed distributions of the breast cancer cases from the four survey areas are shown in Figure 3. Overall, the prevalence of breast cancer varied with significantly over time (Chi-squared = 50.942, p = 0.010). Among the 320 breast cancer cases, 179 (55.94%) were diagnosed within the past three years (see Figure 4), including 78 cases within the last year, 45 cases within the last two years and 56 cases within the last three years. The cases diagnosed within the last three years accounted for 47.0% of cases (39/83) in Hebei Province, 59.7% (92/154) in Shandong Province, 57.5% (27/47) in Jiangsu Province and 58.4% (21/36) in Tianjin.

The demographic characteristics for the breast cancer patients are shown in Table 4, indicating a statistically significant difference in the breast cancer prevalence among different population groups. Differences were seen in various levels of education (p = 0.001), individual annual income (p = 0.005), BMI-body mass index (p<0.001) and breast cancer family history (p<0.001). There were statistically significant differences in the prevalence of breast cancer among some of the variables representing female physiological characteristics (Table 5), including menstrual cycle regularity, number of childbirths, number of abortions/miscarriages, menopausal status, history of benign breast disease, mammary gland hyperplasia, nipple discharge, presence of an accessory breast and nipple retraction. Those physiologic variables not associated with significant differences in breast cancer prevalence included the age at menarche, history of dysmenorrhea and history of oral contraceptive use. Those chronic diseases associated with a significant difference in the distribution of breast cancer cases (Table 6) included cervical cancer, ovarian cysts, diabetes, hypertension and coronary heart disease.

The cases diagnosed within the past two years (n = 123) were used for the 1∶3 matched analysis of factors affecting breast cancer. The average age of the patient group with diagnosed disease was 49.92±9.36 years old, and the average age of the control group was 49.95±9.49 years old; there was no significant difference between the two groups (p = 0.851). In the patient group, 96 cases (78.0%) were from rural areas compared to 272 cases (73.7%) in the control group; there was no statistically significant difference between the two groups (p = 0.337). Other basic demographic characteristics of the two groups are shown in Table 7.

The results of the univariate analysis at the level of α = 0.20 indicated that the breast-cancer-related factors included education level, household annual income, body mass index (BMI), breast cancer family history of primary or secondary relatives, number of miscarriages/abortions, regularity of menstruation, menopausal status at diagnosis, history of benign breast tumor, nipple discharge, soy product consumption, garlic product consumption, insomnia, current life satisfaction, overall life satisfaction, financial status, social status, the behavioral prevention score and the related knowledge score. Multivariate logistic regression analysis (α = 0.10) identified nine variables related to breast cancer: BMI, menopausal status at diagnosis, history of benign breast tumor, family history of breast cancer, history of diabetes, number of miscarriages/abortions, overall life satisfaction, garlic product consumption, financial status and the behavioral prevention score (Table 8 & 9).

## Discussion

This survey is the first large-scale, cross-province epidemiological investigation in China since the 1970s. A total of 320 cases of breast cancer were identified in this survey. The cases diagnosed within the past three years accounted for 67.6% (179/265) of those cases diagnosed within the past ten years and for 55.9% (179/320) of all cases. Further investigation found that the breast cancer cases identified in this study were primarily diagnosed in the 40–45 and 45–50 year age groups (21.6% and 30.3%, respectively), suggesting that the peak of breast cancer incidence in China may be 15–20 years before the peak incidence in Western countries [6]. There were 192 premenopausal cases, accounting for 60% of the identified breast cancer cases, and 128 postmenopausal cases, accounting for 40%. The majority of breast cancer cases are premenopausal in China, which is different from European and American countries, where the majority of breast cancer cases are postmenopausal [7]. This result is consistent with the finding of Li et al, which showed that the premenopausal cases accounted for 62.9% [8]. In our study, both the univariate and multivariate analyses showed that the menopausal status at diagnosis was associated with breast cancer and premenopause was a risk factor of breast cancer (OR: 1.826). In fact, in our study, the premenopausal women accounted for 70.3% among all the surveyed individuals and 60% in the case group, suggesting that more attention should be paid to the premenopausal women. Among the 320 breast cancer cases, 91.6% were diagnosed after the age of 35 and 8.4% were diagnosed before the age of 35, which emphasizes the importance of regular breast examinations for Chinese women over the age of 35 or additional interventions to preemptively prevent breast cancer. On the other hand, this study also indicates the possibility that the age of onset for breast cancer in Chinese women is declining, suggesting that breast cancer prevention should not be neglected for women younger than 35.

Although a large number of studies have been reported on the factors affecting breast cancer in China by both domestic and international researchers, previous results were inconsistent due to the lack of a population-based study of risk factors [8]–[10]. In this study, we found that the average age of menopause for Chinese women was 48.8 years old and that the average age of menarche was 15.4 years old. Only univariate logistic analysis showed that irregular menstruation could increase the risk of breast cancer (OR: 1.743), which was consistent with the results of prospective studies by other international researchers [11]–[13]. The age of menarche of Chinese women is two to three years later than the age of menarche in women from the US and European countries. The characteristics of late menarche, early menopause and short menstrual cycles in Chinese women may partially explain the low incidence of breast cancer in China. Childbirth, especially with full-term pregnancy and associated breastfeeding [14], has been generally considered an important protective factor against breast cancer. Multiple childbirths could also reduce the risk of breast cancer [12], [15]. Both univariate and multivariate logistic regression analyses showed a statistically significant correlation between miscarriage/abortion and breast cancer, and the ORs were 1.644 and 1.497, respectively. Internationally, there is no conclusive evidence on the relationship between miscarriage/abortion and breast cancer. Some of the studies have supported the correlation between abortion and breast cancer [16]–[18], while others have indicated no apparent relationship [19]–[21]. Also in this study, there were no relationships identified between breast cancer and some factors that have been previously shown to be related to breast cancer, such as age at menarche, menstrual years, menopause, age at first childbearing, full-term pregnancy and breastfeeding. It may be explained by the following reasons: Traditionally, Chinese women are requested for childbirth and breastfeeding, and they enjoy taking these responsibilities. In our study, 97.2% of the subjects had given birth to at least one child and 78.6% had breastfed for at least 12 months; few women have never given birth or breastfed. Chinese couples were permitted one or two children only since the implementation of birth control policies beginning in 1970s. Therefore, it is hard to explore the relationship between multiple deliveries and breast cancer. Neither univariate nor multivariate analysis showed any statistical significance when compared with other factors. Wang et al. [22] conducted a case-control study on breast cancer risk factors in six cities in China, including Beijing, Tianjin and Shanghai, which also showed inconsistent conclusions.

Breast cancer displays an apparent familial tendency. Univariate analyses were used to study the primary and secondary family history of breast cancer, as well as the unclassified family history of breast cancer. The results demonstrated statistical correlations between a family history of breast cancer and the incidence of breast cancer. The unclassified family history of breast cancer was selected for the multivariate logistic analysis, and the results also indicated that family history of breast cancer is a risk factor for breast cancer (OR: 5.438), which is consistent with the results from other international studies [23], [24]. Patients with benign breast diseases have a higher risk of breast cancer than healthy individuals [25], [26]. The results from the present study also indicated a close correlation between the history of benign breast tumors and breast cancer (OR: 3.357), suggesting that a history of benign breast diseases is an important risk factor for breast cancer in women. These results are consistent with results from the cohort studies by Fang et al. [27] and Colep [28].

This study discovered a correlation between history of diabetes and breast cancer (OR: 3.556). Previous studies indicated that type 2 diabetes is a risk factor for breast cancer [29]. The results from the meta-analysis showed diabetes and the risk of breast cancer revealed that diabetes increased the risk of breast cancer by 20% [30]. With the exception of the case-control study by Liao et al. [31], which indicated that diabetes was a risk factor for breast cancer, no other reports were found on this relationship between diabetes and breast cancer in Asian women. Diabetes, especially type 2 diabetes, is closely related to obesity. In China, 30% of type 2 diabetes patients are obese. Diabetes together with obesity may increase the risk of breast cancer [32].

This study found that a high BMI had a statistically significant correlation with breast cancer (OR: 1.528), which is consistent with the results from the previous studies [33], [34]. With the changes in lifestyle and improvements in living conditions in China, overweight and obese people account for a large proportion of the Chinese population. The epidemiological survey of diabetes conducted in 2008 indicated that the prevalence of diabetes was 11.6% in urban populations and 10.6% in women. One study reported that 36.97% of the female population was overweight and that 15.39% was obese [35]. The association between the increasing incidence of breast cancer and the rapid growth of the diabetic and obese population requires further in-depth investigation. A healthy lifestyle and preventing obesity and diabetes are important steps in the prevention of breast cancer, especially in China, given the rapid increase in the overweight and obese population [36].

The present study used the cumulative score of twelve items to describe the overall life satisfaction and the cumulative score of six items to assess current life satisfaction. The scores were classified into two types, high and low scores. High scores indicated low life satisfaction or dissatisfaction, whereas low scores indicated high life satisfaction. The results of this study indicated a positive correlation between low overall life satisfaction (or dissatisfaction) and breast cancer (OR: 2.196), indicating that psychological factors may be associated with breast cancer. Therefore, psychological intervention should be considered in the comprehensive prevention of breast cancer in the population.

**Table 8:** Multivariate logistic analysis of breast cancer-related factors among women.

Factors	Characteristics		P	OR (95% CI)
	Unfavorable	Favorable		
Benign breast disease history	yes	no	0.029	3.357 (1.131–9.969)
Diabetes Mellitus	yes	no	0.069	3.556 (0.904–13.994)
Garlic consumption	infrequent	frequent	0.084	1.231 (0.972–1.559)
Economic Status	low	high	0.104	1.500 (0.920–2.446)
Times of miscarriage	> = 1	0	0.043	1.497 (1.014–2.211)
Family history of breast cancer	yes	no	0.008	5.438 (1.553–19.004)
Global quality of life satisfaction	low	high	0.001	2.196 (1.355–3.556)
BMI	> = 24.0	<24.0	0.016	1.528 (1.083–2.155)
Menstrual status	Premenopausal	Postmenopausal	0.052	1.826(0.995–3.350)
Healthy behavioral prevention score	high	low	0.000	3.556 (1.880–6.728)

**Table 9:** Results of population-based multivariate Logistic regression analysis.

	B	S.E.	Wald	P	OR	OR95%CI	
History of benignbreast tumors	2.436	0.212	131.754	0.000	11.431	7.541	17.328
Family history	1.599	0.234	46.569	0.000	4.946	3.125	7.828
Age assignment	0.569	0.049	136.951	0.000	1.767	1.606	1.944
Times of abortion	0.567	0.119	22.892	0.000	1.763	1.398	2.225
Overall lifesatisfaction	0.520	0.115	20.499	0.000	1.682	1.343	2.107
BMI	0.428	0.087	24.248	0.000	1.535	1.294	1.820
Bean products	0.263	0.068	15.114	0.000	1.300	1.139	1.484
Dairy products	−0.164	0.053	9.551	0.002	0.848	0.765	0.942

The present study has its limitations. First, only 25- to 70-year-old women of the Han ethnic group in Eastern China were selected as the survey targets; therefore, our study results have limitations for extrapolation. Second, the cases used for risk factor analysis were those diagnosed within the past two years. However, it may introduce prevalence-incidence bias (Neyman bias). Third, age is considered as an important risk factor for breast cancer; however, in the present study, we matched the cases according to both age and region to control for confounding factors. Thus, the correlation between age and breast cancer risk was not investigated in this study.

In conclusion, these findings suggest that attention should be focused on the incidence of breast cancer among premenopausal women older than 35. The incidence of breast cancer is associated with biological, behavioral and social factors, and comprehensive measures should be taken to prevent the occurrence of breast cancer.
